# Development and testing of a predictive model of symptoms for pain in community-dwelling frail older people in palliative care

**DOI:** 10.1186/s12904-025-01888-y

**Published:** 2025-10-14

**Authors:** Suzan van Veen, Hans Drenth, Hans Hobbelen, Wim Krijnen, Everlien de Graaf, Evelyn Finnema

**Affiliations:** 1 Health Science-Nursing Science, University of Groningen, University Medical Center Groningen, Groningen, 9713 GZ the Netherlands; 2 ZuidOostZorg, Organization for Elderly Care, Drachten, the Netherlands; 3 Research group Healthy Ageing, Allied Health Care and Nursing, Hanze University of Applied Sciences, Groningen, the Netherlands; 4 FAITH Research, Groningen, Leeuwarden, the Netherlands; 5 Department of Primary and Long-term Care, University of Groningen, University Medical Center Groningen, Groningen, the Netherlands; 6 Department of General Practice and Elderly Care Medicine, University Medical Center Groningen, University of Groningen, Groningen, the Netherlands; 7 Undergraduate School of Science and Engineering, Faculty of Science and Engineering, University of Groningen, Groningen, the Netherlands; 8 Center of Expertise Palliative Care Utrecht, Dept of General Practice and Nursing Science, Julius Center for Health Sciences and Primary Care, University Medical Center Utrecht, Utrecht, the Netherlands

**Keywords:** Palliative care, Frail elderly, Pain, Symptom assessment, Pain management, Clinical decision rules

## Abstract

**Background:**

Pain assessment is a necessary step in pain management in older people in palliative care. In older people, pain assessment can be challenging due to underreporting and atypical pain manifestations by other distressing symptoms. Anxiety, fatigue, loss of appetite, nausea, insomnia, dyspnoea, and bowel problems correlate with pain in palliative care patients. Insight into these symptoms as predictors may help to identify the underlying presence of pain. This study aimed to develop and test a prediction model for pain in community-dwelling frail older people in palliative care.

**Methods:**

In this cross-sectional observational study, community-care nurses from multiple organizations across the Netherlands included eligible patients (life expectancy < 1 year, aged 65+, community-dwelling and frail). The outcome pain and symptoms were assessed by means of the Utrecht Symptom Diary. Also, demographic and illness information, including relevant covariates age, sex and living situation, was collected. Multivariable logistic regression and minimum Akaike Information Criterion(AIC) were used for model development and Receiver Operating Characteristics(ROC)-analysis for model performance. Additionally, predicted probability of pain are given for groups differing in age and sex.

**Results:**

A total of 157 patients were included. The final model consisted of insomnia(Odds Ratio[OR] = 2.13, 95% Confidence Interval[CI] = 1.01–1.30), fatigue(OR = 3.47, 95% CI = 1.11–1.43), sex(female)(OR = 3.83, 95% CI = 2.11–9.81) and age(OR=-1.59, 95% CI = 0.92–1.01) as predicting variables. There is an overall decreasing trend for age, older persons suffer less from pain and females have a higher probability of experiencing pain. Model performance was indicated as fair with a sensitivity of 0.74(95% CI = 0.64–0.83) and a positive predictive value of 0.80(95% CI = 0.70–0.88).

**Conclusions:**

Insomnia and fatigue are predicting symptoms for pain, especially in women and younger patients. Further testing of the model in external cohorts is needed before clinical adoption.

## Introduction

Unrelieved pain is a common problem in palliative care patients [1]. Worldwide approximately 25 million people die in pain each year [2]. Pain is consistently found as an important symptom for one-third of older people in palliative care [3]. In the concept of total pain, the central belief is that pain emerges from both physical and nonphysical sources [4]. The concept recognizes that palliative care patients will also suffer from distressing symptoms other than pain. Therefore it is necessary to assess and manage all potential sources of additional distress [5]. Pain rarely occurs in isolation and co-occurring symptoms appear to have synergistic associations with patients’ treatment outcomes, prognosis, functional status, and quality of life [6]. Cohen and Mount note a bidirectional relationship: not only does pain affect all aspects of the person, but all aspects of the person can contribute to the perception of pain [7]. Older people who are frail exhibit an increased vulnerability to pain. Their frailty status is associated with an increased risk of experiencing persistent, acute or combined forms of pain. Given the impact of pain and other associated symptoms on frail older peoples’ health, there is value in a palliative care approach [8].

According to the World Health Organization (WHO), evidence has shown that older people suffer unnecessarily because of widespread underassessment and undertreatment of health-related problems [3]. Pain is often underreported in older people partly due to their beliefs that pain is a normal consequence of ageing [9]. It is not apparent whether these “age normative” beliefs are influential on other distressing symptoms in the last stages of life. Effective pain assessment in older people includes challenges such as the underreporting of pain on the part of the patients, proper assessment of pain, and atypical manifestations of pain (through experiencing other distressing symptoms) [10].

The atypical manifestations and the bidirectional relationship of symptoms with pain can contribute to the perception of experiencing pain in older people. Predicting underlying pain by determining other symptoms as predictors may help to identify pain and challenge underreporting and consequently undertreatment of pain in older people. A variety of symptoms are correlated with pain in palliative care patients; nausea [11], anxiety [12], fatigue [13], loss of appetite [12], insomnia [14], dyspnoea [15], and bowel problems [16].

Little is known about these univariate associations with older palliative care patients with a single exception [12]. The associations found could differ from the population of community-dwelling frail older people in palliative care due to age-related decline, comorbidities, and high mortality.

A lack of evidence exists regarding predicting underlying experiences of pain in community-dwelling frail older people in palliative care. In clinical practice, a prediction model can help to identify pain and open the possibility of discussing adequate pain management with the patient and/or relatives.

The aim of this study is to develop a prediction model for pain and test model performance in community-dwelling frail older people in palliative care.

## Methods

This cross-sectional observational study assessed the predicting variables (symptoms and covariates) and the outcome variable (pain) simultaneously for each patient. The study took place from February 2021 to September 2023. Transparent Reporting of a multivariable prediction model for Individual Prognosis Or Diagnosis (TRIPOD) Checklist for prediction model development was used to facilitate reporting of the study [17].

### Sample

Data were collected from community-dwelling frail older people in palliative care from thirteen community-care organizations across the Netherlands. These community-care organizations provide long-term care or specialized care at home. For this study, a convenience sampling method was used to select eligible patients. The eligibility criteria were: having a life expectancy of less than one year, aged 65 years or older, living at home, receiving assistance from home-care nursing, screened as frail (score = 4–15) based on the Groningen Frailty Indicator (GFI)-questionnaire [18], and able to self-assess and communicate their symptoms. Estimated life expectancy was determined by community-care nurses based on the valid and reliable ‘surprise question’: “Would I be surprised if this patient were to die in the next twelve months?” [19], which is highly effective in predicting patients in high need of palliative care [19]. Patients with a diagnosis of dementia or mild cognitive impairment were excluded to ensure the reliability of symptom intensity scores. The sample size was determined a priori, computed with G*power version 3.1.9.4. using a power 0.90 and significance level 0.05, resulting in an estimated sample size of 168 patients [20]. The effect size was calculated using linear multiple regression with Cohen’s f2 of 0.114 based on the correlations of the associated symptoms of the study aim known in palliative care patients [20].

### Data collection

The Utrecht Symptom Diary (USD) was used to assess the intensity of predicting symptoms as well as outcome variable pain [21]. The USD is a validated Dutch version of the Edmonton Symptom Assessment System (ESAS) to self-assess the eleven most prevalent symptoms in cancer patients [21, 22]. The severity of symptoms at the time of assessment was rated from zero to ten on a Numerical Rating Scale (NRS), in which zero means the least and ten is the most possible symptom severity [22]. Covariates such as sex, age, GFI-score and primary diagnosis, were collected from the Case Report Form (CRF). The availability of informal care was asked and entails someone who provides unpaid help to a relative needing support.

### Procedures

Multiple community-care nurses from different organizations assisted in the data collection process. Through networking efforts, they voluntarily helped with data collection for this study, and new nurses were continuously approached and trained in eligibility criteria and data collection. Each participating community-care nurse checked the eligibility of their patients with the use of the study protocol or in collaboration with the main researcher (SvV). Eligible patients were invited to participate and provided with study information. Before data collection began they signed the informed consent form. Data was collected cross-sectionally on the hard-copy questionnaires CRF and USD. The community-care nurses completed the CRF in consultation with the patients, while patients self-assessed their symptoms on the USD. Collected data per patient was sent to the main researcher by secure email. Before entering collected data into the database, a respondent identification number was assigned by the main researcher (SvV) for each case.

### Data analysis

Descriptive analysis was used for patient characteristics (mean and SD or N(%)).The prevalence of the selected symptoms, defined as USD scores larger or equal to one, is reported to describe the sample, distinguishing between the absence and presence of a symptom. Only for descriptive purposes of the sample, the frequencies of symptom scores equal to and greater than three are reported as they are seen as clinically relevant [21]. The proportion of missing values for the variables involved was computed. Missing values of the variables with less than 5% missing were imputed by the variable means or random categories. The outcome-variable pain was dichotomized into PresPain indicating the presence or absence of pain according to scores larger or equal to one.

A multivariable logistic regression model was created with the dichotomized USD pain score as the outcome variable and continuous symptom scores of anxiety, fatigue, loss of appetite, insomnia, nausea, dyspnoea, and bowel problems as predictor variables. Covariates (age, sex, and living situation) were selected based on known relevance [23, 24]. The predicting symptoms and covariates were selected according to the minimum Akaike Information Criterion(AIC), which results in the best predictive model [25]. The Odds Ratios (OR) and effect plots give the effects of the selected variables on the presence of pain (PresPain). Additionally, several figures will be given predicting the underlying presence of pain for groups differing in age and sex with prediction lines and confidence bands [26].

To determine the predictive accuracy of the final model, the Receiver Operating Characteristics (ROC)-analysis was used to determine the Area Under the ROC Curve (AUC). An AUC of ≥ 0.80 indicates good and an AUC between 0.70 and 0.80 fair accuracy [27]. The sensitivity, specificity, positive predictive value (PPV), negative predictive value (NPV) and their confidence intervals are reported [28]. Statistical significance (two-sided) was set at *p* ≤ 0.05. All analyses were conducted using Statistical Package for the Social Sciences (SPSS) version 23.0 and R version 2.0.60 [29, 30].

### Ethics

Patients provided written consent to use their collected data for scientific purposes. Regulations of the General Data Protection were followed [31]. The study was conducted according to the Declaration of Helsinki (the latest version WMA General Assembly 2013) [32]. A non-WMO (the Medical Research Involving Human Subject Act) statement was granted by the Ethics Committee of the University Medical Center Groningen [registration number: 202100021] [33].

## Results

### Respondents

In total, 157 patients were enrolled in this study. Of these patients, 38.2% were male and the mean age was 83.4 (SD ± 8.4) of whom 60.5% lived alone and 35.6% lived with a partner or relatives. The primary informal caregiver was in 28% living with the patient and 58% non-residential informal caregiver. The primary diagnosis was predominantly cardiovascular diseases (33.1%), followed by cancer (22.9%) and diseases of the nervous- and sensory system (15.3%). The mean frailty score was 7.5 (SD ± 2.5). All patient characteristics are presented in Table 1.

**Table 1:** Patient characteristics of the respondents (*N* = 157)

**Characteristic**	**Values**
Sex[male], N (%)	60 (38.2%)
Age in years, mean ± SD	83.4 ± 8.4
Score on Groningen Frailty Indicator, mean ± SD	7.5 ± 2.5
Living situation, N (%)	
• Alone	95 (60.5%)
•With a significant other	52 (33.1%)
•With other relatives	4 (2.5%)
•Missing observation	6 (3.8%)
Availability informal caregiver, N (%)	
•Non-residential informal caregiver	91 (58%)
•Living with an informal caregiver	44 (28%)
•No availability of informal care	22 (14%)
Primary diagnosis, N (%)	
•Cancer [total]	36 (22.9%)
-Urological	12 (7.6%)
-Gastrointestinal	11 (7.0%)
-Lung	5 (3.2%)
-Brain	2 (1.3%)
-Gynaecological	2 (1.3%)
-Skin	1 (0.6%)
-Other primary cancer sites	3 (1.9%)
•Disease of the cardiovascular system	52 (33.1%)
•Disease of the nervous- and sensory system	24 (15.3%)
•Disease of the respiratory system	18 (11.5%)
•Disease of the digestive system	6 (3.8%)
•Other primary diagnosis	19 (12.1%)
•Missing observation	2 (1.3%)
Use of pain medication[yes], N (%)	107 (68.2%)
Type of pain medication[use=yes], N (%)	
•Non-opioids	72 (67.3%)
•Opioids	15 (14.0%)
•Both	20 (18.7%)

### Prevalence and intensity of selected symptoms

The prevalence of pain was 61.8% with a mean intensity score of 3.4 (SD ± 3.3). Most uncommon symptom was nausea: 19.1% with a score of one or higher, and 10.8% with a score of ≥ 3. Most prevalent symptom was fatigue (84.7%) and had the highest mean intensity score (5.1 ± 3.0). The prevalence of USD symptoms was larger than 50% for six out of eight in total.

**Table 2:** Intensity (mean and SD), prevalences and clinically relevant prevalences based on the selected USD symptoms (*N* = 157)

USD symptoms	Mean (± SD)	USD* ≥ 1 (*N*)	%	USD* ≥ 3 (*N*)	%
Pain	3.4 ± 3.3	97	61.8	82	52.2
Insomnia	3.4 ± 3.1	103	65.6	87	55.4
Loss of appetite	3.0 ± 3.1	99	63.1	76	48.4
Bowel problems	3.6 ± 3.2	116	73.9	86	54.8
Nausea	0.7 ± 1.7	30	19.1	17	10.8
Dyspnoea	2.6 ± 3.2	80	51.0	61	38.9
Fatigue^#^	5.1 ± 3.0	133	84.7	118	75.2
Anxiety	1.7 ± 2.6	60	38.2	42	26.8

*USD is an abbreviation of Utrecht Symptom Diary

^#^Missing value on 1 case (0.6%)

### Model development and specifications

Of all data points, 0.64% were missing. Of the explanatory variables the missing data was imputed so that all available cases could be included for further analysis (*N* = 157). The model was developed using multivariable logistic regression and minimum AIC model selection, revealing insomnia (OR = 2.13, 95% Confidence Interval(CI) = 1.01–1.30) and fatigue (OR = 3.47, 95% CI = 1.11–1.43) were independent predicting symptoms for pain, after correction for age and sex. Sex (female)(OR = 3.83, 95% CI = 2.11–9.81) and age (OR=−1.59, 95% CI = 0.92–1.01) were the predicting covariates. Specifications of the final model after minimum AIC are presented in Table 3.

**Table 3:** Logistic regression estimates after minimum AIC, standard errors, p-values, odds ratios (Exp(B)), and their 95% confidence intervals

Variables	Regression coefficient B	Standard error	*p*-value	Exp(B)	95% Confidence interval for Exp(B)	
					lowest	highest
Constant	1.070	1.919	0.577	0.56	0.07	132.59
Age	−0.036	0.023	0.111	−1.59	0.92	1.01
Sex(female = 1)	1.493	0.390	0.000	3.83	2.11	9.81
Insomnia	0.135	0.063	0.033	2.13	1.01	1.30
Fatigue	0.226	0.065	0.000	3.47	1.11	1.43

**Fig. 1:**
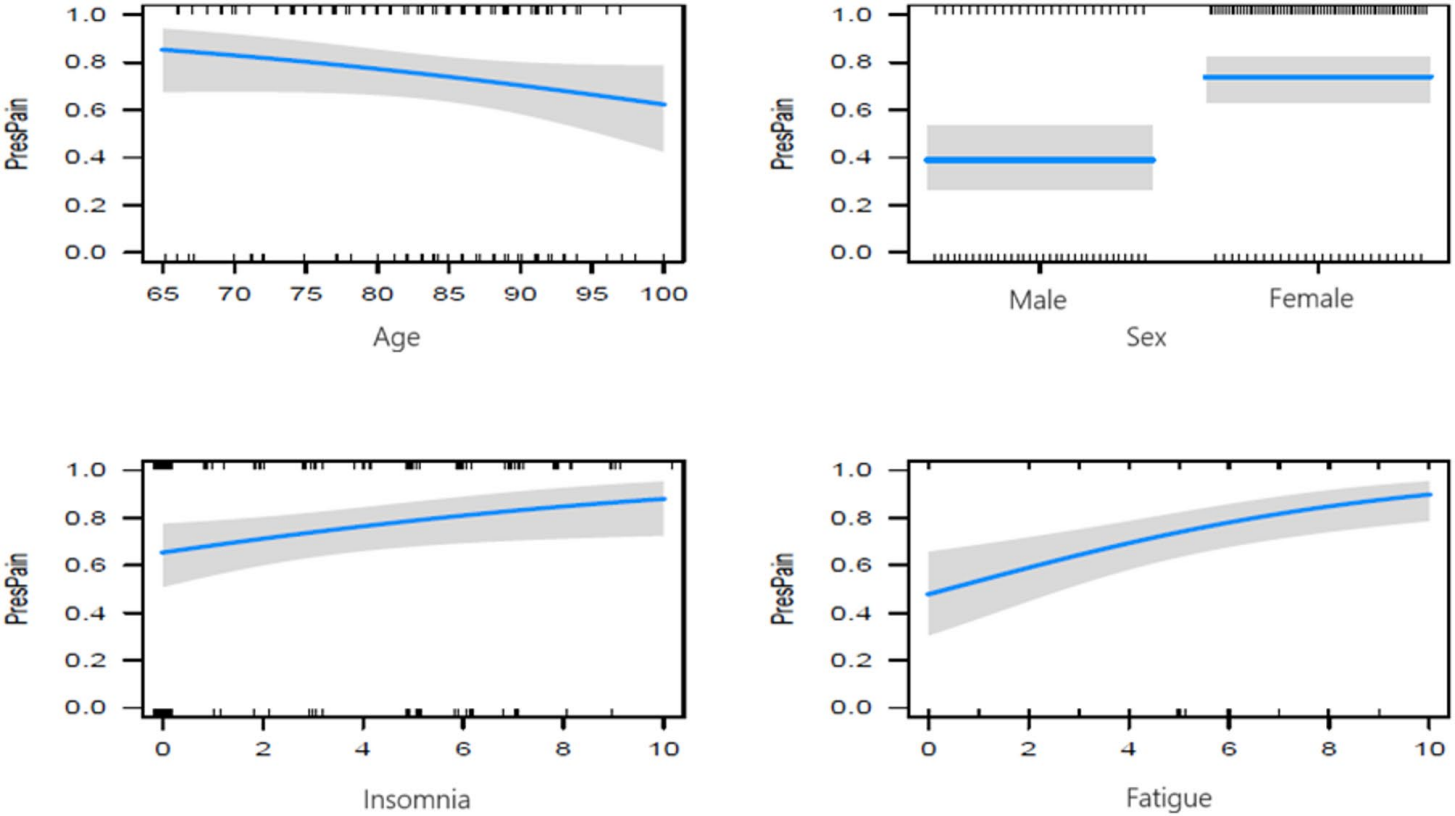
Effects from logistic regression of age, sex, insomnia and fatigue on predicted probability of pain (vertically)

**Fig. 2:**
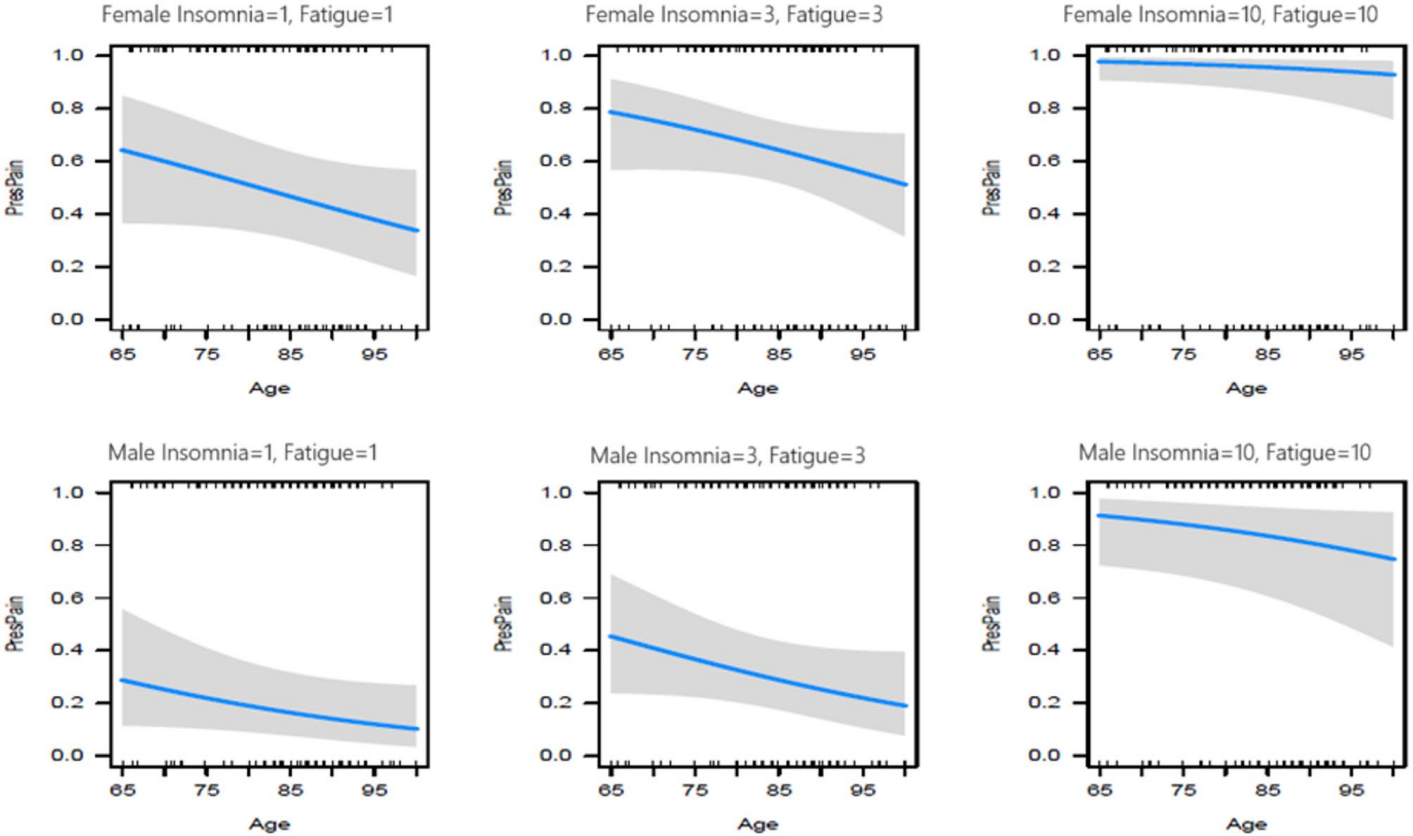
Predicted probability of pain (vertically) from logistic regression depending on age and insomnia and fatigue across sex

The effects of Age and Sex on the probability of the person experiencing pain is visualized with prediction lines and confidence bands in Fig. 1. It can be observed that there is a slight decrease in pain experience with increasing Age. Persons with a probability larger than the cut-off of 0.599 are best predicted to have pain by the model. How the probability of pain depends on Age, Sex, Insomnia and Fatigue is detailed in Fig. 2. There is an overall decreasing trend for Age, older persons suffer less from pain. For low USD values, both females and males are below the cut-off value of 0.599, whereas for large USD values both are above. For USD values equal to 3 females younger than 85 years of age are above the cut-off value. The figure illustrates the difference in the effect of low versus high USD levels on the probability of pain. In particular, the effect for males is more drastic than for females.

### Model performance

The final model resulted in 42 true negative, 25 false negative, 18 false positive and 72 true positive predictions causing the overall percentage of correct predictions equal to 80%. The estimated sensitivity is 0.74 (95% CI = 0.64–0.83), the specificity 0.70 (95% CI = 0.57–0.81), the positive predictive value 0.80 (95% CI = 0.70–0.88) and the negative predictive value 0.63 (95% CI = 0.50–0.74). The AUC was calculated at 0.768 (95% CI = 0.692–0.843).

## Discussion

### Summary of the results

This study aimed to determine whether the seven symptoms anxiety, fatigue, loss of appetite, insomnia, nausea, dyspnoea, and bowel problems are predictors for experiencing pain in community-dwelling frail older people in palliative care. This study found that insomnia and fatigue are statistically significantly independent predicting symptoms for pain after correcting for sex and age. The analysis gave age as a predicting covariate, although not significant did give an effect in the final model (see Fig. 1).

The AUC showed fair predictive accuracy. The estimated OR of insomnia indicated that persons experiencing insomnia one score higher have a 2.13 times higher chance of experiencing pain. For fatigue, the OR is 3.47 times higher. The OR of sex indicated that females have a 3.83 times higher chance of experiencing pain than males. Although not statistically significant, the overall decreasing trend for age, where older persons suffer less from pain, is seen in the OR of −1.59.

### Reflection on the study results

Almost two-thirds of the sample in this study recorded the presence of pain (61.8%). This was similar to other studies of pain experienced in older people with a life expectancy of less than one year with a prevalence of 66.3% [34] and a prevalence range of 57–88% [35].

Insomnia and fatigue were found to be significant and relevant predictors for the presence of pain. Pain processing happens in the insula and the somatosensory cortex of the brain. The increased involvement of the anterior insula was negatively associated with insomnia [36] and is involved in the experiencing of fatigue [37]. The insula integrates sensory with emotional and cognitive processes and is involved in aversive motivational salience [38]. Salience processing is often associated with the extension of certain sensory inputs, such as the interoceptive stimuli of pain [38]. The processing of these symptoms within the insula might explain the predictability of pain.

The above effect of sex indicated that women are at a higher risk for common pain conditions in comparison to men [39]. Hormonal factors are thought to explain sex differences in pain perception as these regulate the cortical processing of pain-related stimuli [39]. The decreasing effect of age indicated that older people suffer less from pain. This might be explained by a change of the peripheral nerves and receptors with ageing resulting in different pain sensitivity [40] and, the generational differences in pain beliefs and attitudes that may occur across age groups [41]. Not only can these biological factors explain the prediction of pain by these symptoms and covariates, also psychosocial factors may have an influence. For example, as a psychological factor, a change in cognitive beliefs or coping skills. With age, cognitive changes can challenge an older person’s ability to accurately recall and report past symptom burden experiences, which can lead to inconsistencies in pain reporting over time [42]. Maladaptive cognitions about sleep and pain can lead to inadequate coping of seeking pain management [43].

To consider the social factors of relating pain to its predictors, it is needed to think about the decreasing social networks size and quality with increasing age [44]. Therefore, making it indirectly more challenging to seek the right support for pain recognition and management. The different dynamics in the living situation can also result in differences between sex, for example specific behavioral factors such as care(giving) responsibilities or cultural/social norms [45].

### Strengths and limitations

A strength of this study was that the analysis was run on a near-complete dataset. Less than 1% of all data points (20 of 3140 data points, 0.64%) were missing and of the explanatory variables less than 1% of data points (nine of 1570 data points, 0.57%) were missing. These data points were considered to be missing at random. Another strength is that the developed prediction model had fair to high sensitivity (0.74) and specificity (0.70) resulting in a high proportion of positives correctly identified (0.80) as experiencing pain. With a sensitivity of 0.74, the model correctly identifies 74% of older palliative care patients with pain (≥ 1). Given the high pain prevalence (61.8%), this supports its value in improving recognition, especially considering the underreporting in this population. The distribution in the prevalence of pain in this sample improves its ability to distinguish between both outcomes accurately. The empirical findings of this study are supported by the clinical perspective in other palliative care populations, where the symptom of fatigue [46], and insomnia co-occur with pain [47]. It is of importance to address both pain, fatigue, and insomnia concurrently, especially in the risk groups identified in this study in the population of older palliative care patients.

This study also had some limitations. Part of the inclusion criteria was to assess the palliative phase based on the surprise question. Some community-care nurses gave the feedback that they found the palliative phase (life expectancy < 6 months), terminally ill (life expectancy < 2 weeks) or end-stage diseases easier to identify. This may have introduced some selection bias due to underestimation resulting in a more frail group than intended. However, the descriptive statistics of the sample do not substantiate this bias. In this study, the sample consisted of older palliative care patients who were identified as frail based on the GFI-criteria. The broad range of possible frailty scores (GFI 4–15), may influence the applicability of a general predictive model for pain. However, given that underreporting and under recognition of pain are widespread issues in this population, our model aims to provide an initial step toward improving pain identification. It may certainly be beneficial in future research to include specificities of patient groups in a generic model. Additionally, the convenience sample strategy may have resulted in some selection bias. The distributions of characteristics do not imply this bias.

The sample size was 157 instead of the 168 required by the sample size calculation, as a result, this study might be underpowered. Therefore, the confidence intervals around some estimates may be wider than if the full sample had been achieved. Future studies with larger cohorts could improve the precision of predictive estimates.

It may be noted that the USD was validated among cancer patients [21]. This instrument is widely used in the Netherlands as the multidimensional symptom assessment instrument within palliative care. Moreover, the current population overlaps with the cancer patients, and it seems reasonable to assume that the results of the validation study are generalizable to the current population. The best fit model was selected based on the minimum AIC and the model performance was tested on the same dataset, therefore the diagnostics of the performance may have resulted in some overestimation. For this population, this analysis is exploratory, and incorporating changes over time is an addition for the further development of the predictive model.

### Recommendations for practice

The use of the USD or any other symptom-burden measurement instrument, in combination with the prediction model, can be helpful in identifying pain in older palliative care patients. Insomnia and fatigue are predicting symptoms for pain, especially in women and in younger patients. The identified risk groups demonstrate a high predictive accuracy of the underlying pain experience. However, challenges such as the variability in symptom perception and tolerance, the timing and frequency of assessments, and the role of palliative care professionals in symptom recognition and management remain critical considerations.

Future research should focus on the external validation of this model to support in the early identification of pain in older palliative care patients and enhance its predictive accuracy. Once validated, the model could be incorporated into decision-support tools in palliative care.

The results of this model are based on the population of community-dwelling frail older people with a life expectancy of less than one year. In this final stage in life, it is, for the most part, still possible to communicate with patients and/or relatives about advance care planning and their desired pain management. Identifying the underlying presence of pain is therefore an essential part of care. Therefore, symptom assessment, especially for the risk groups identified, can help the palliative care professional in the management of pain.

## Conclusions

This study showed insomnia and fatigue as statistically significant independent predictors for the presence of pain in community-dwelling frail older people with a life expectancy of less than one year. The final prediction model presented has sex and years of age as additional effects on the presence of pain. The predictive accuracy of the model for the presence of pain was fair with a high positive predictive value of 0.80. The current study identified several subgroups which are highly at risk for underlying presence of pain. Further testing of the model in external cohorts is needed before clinical adoption.

## Data Availability

The dataset used and analyzed during the current study is available from the corresponding author on reasonable request.

## Abbreviations

AIC: Akaike information criterion
AUC: Area under the curve
CI: Confidence interval
CRF: Case report form
ESAS: Edmonton symptom assessment system
GFI: Groningen frailty indicator
N: Number of proportion
NPV: Negative predictive value
NRS: Numerical rating scale
OR: Odds ratio
PPV: Positive predictive value
ROC: Receiver operating characteristics
SD: Standard deviation
SPSS: Statistical package for social sciences
TRIPOD: Transparent reporting of a multivariable prediction model for individual prognosis or diagnosis
USD: Utrecht symptom diary
WHO: World health organization
WMO: Wet medisch-wetenschappelijk onderzoek met mensen (Dutch) [Medical research involving Human Subject Act

## References

[1] Klint Å, Bondesson E, Rasmussen BH, Fürst CJ, Schelin MEC. Dying with unrelieved pain—prescription of opioids is not enough. J Pain Symptom Manage. 2019;58(5):784-e7911. 31319106 10.1016/j.jpainsymman.2019.07.006

[2] Bhatnagar S, Gupta M. Integrated pain and palliative medicine model. Ann Palliat Med. 2016;5(3):196–208. 27334349 10.21037/apm.2016.05.02

[3] World Health Organisation Europe. Davies E, Higginson IJ, eds. Better palliative care for older people. Copenhagen, WHO Regional Office for Europe. Palliative care for older people: better practices 2004:1–40.

[4] McPherson CJ, Hadjistavropoulos T, Lobchuk MM, Kilgour KN. Cancer-related pain in older adults receiving palliative care: patient and family caregiver perspectives on the experience of pain. Pain Res Manag. 2013;18(6):293–300. 23957019 10.1155/2013/439594 PMC3917792

[5] Platt M. Pain challenges at the end of life - Pain and palliative care collaboration. Rev Pain. 2010;4(2):18–23. 26527076 10.1177/204946371000400205 PMC4590058

[6] Dong ST, Butow PN, Costa DSJ, Lovell MR, Agar M. Symptom clusters in patients with advanced cancer: a systematic review of observational studies. J Pain Symptom Manage. 2014;48(3):411–50. 24703941 10.1016/j.jpainsymman.2013.10.027

[7] Cohen SR, Mount BM. Pain with life-threatening illness: its perception and control are inextricably linked with quality of life. Pain Res Manag. 2000;5(4):271–5.

[8] Booker SS, Bartoszczyk DA, Herr KA. Managing pain in frail elders. Am Nurse Today 2016;11(4):1-9 . PMC5179031 28018518

[9] Hofland SL. Elder beliefs: blocks to pain management. J Gerontol Nurs. 1992;18(6):19–23. 1602111 10.3928/0098-9134-19920601-05

[10] Cavalieri TA. Management of pain in older adults. J Am Osteopath Assoc. 2005;105(3 Suppl 1):12. 18154193

[11] Wilson KG, Chochinov HM, Allard P, Chary S, Gagnon PR, Macmillan K, et al. Prevalence and correlates of pain in the Canadian National palliative care survey. Pain Res Manag. 2009;14(5):365–70. 19862371 10.1155/2009/251239 PMC2779154

[12] Black B, Herr K, Fine P, Sanders S, Tang X, Bergen-Jackson K, Titler M, Forcucci C. The relationships among pain, nonpain symptoms, and quality of life measures in older adults with cancer receiving hospice care. Pain Med. 2011;12(6):880–9. 21539700 10.1111/j.1526-4637.2011.01113.x PMC3117028

[13] Stone P, Hardy J, Broadley K, Tookman AJ, Kurowska A, A’Hern R. Fatigue in advanced cancer: a prospective controlled cross-sectional study. Br J Cancer. 1999;79(9–10):1479–86. 10188894 10.1038/sj.bjc.6690236 PMC2362706

[14] Delgado-Guay M, Yennurajalingam S, Parsons H, Palmer JL, Bruera E. Association between self-reported sleep disturbance and other symptoms in patients with advanced cancer. J Pain Symptom Manage. 2011;41(5):819–27. 21306864 10.1016/j.jpainsymman.2010.07.015

[15] Tanaka K, Akechi T, Okuyama T, Nishiwaki Y, Uchitomi Y. Factors correlated with dyspnea in advanced lung cancer patients: organic causes and what else? J Pain Symptom Manage. 2002;23(6):490–500. 12067773 10.1016/s0885-3924(02)00400-1

[16] Clark K, Smith JM, Currow DC. The prevalence of bowel problems reported in a palliative care population. J Pain Symptom Manage. 2012;43(6):993–1000. 22651945 10.1016/j.jpainsymman.2011.07.015

[17] Collins GS, Reitsma JB, Altman DG, Moons KGM. Transparent reporting of a multivariable prediction model for individual prognosis or diagnosis (TRIPOD): the TRIPOD statement. BMJ. 2015;350:g7594. 25569120 10.1136/bmj.g7594

[18] Slaets J. Groningen Frailty Indicator: Instructie ontleend aan werkwijzen screening op kwetsbaarheid. Available from: https://www.pallialine.nl/uploaded/docs/Kwaliteitskader_pz/Meetinstrument_GFI.pdf?u=1PpZQ+. Accessed 6 Nov 2020.

[19] Veldhoven CMM, Nutma N, De Graaf W, Schers H, Verhagen CaHHVM, Vissers KCP, Engels Y. Screening with the double surprise question to predict deterioration and death: an explorative study. BMC Palliat Care. 2019;18(1):118. 31881958 10.1186/s12904-019-0503-9 PMC6935168

[20] Faul F, Erdfelder E, Lang A, Buchner A. G*power 3: a flexible statistical power analysis program for the social, behavioral, and biomedical sciences. Behav Res Methods. 2007;39(2):175–91. 17695343 10.3758/bf03193146

[21] Baan FH, Koldenhof JJ, Nijs EJ, Echteld MA, Zweers D, Hesselmann GM, et al. Validation of the Dutch version of the Edmonton symptom assessment system. Cancer Med. 2020;9(17):6111–21. 32643871 10.1002/cam4.3253 PMC7476846

[22] Bruera E, Kuehn N, Miller MJ, Selmser P, Macmillan K. The edmonton symptom assessment system (ESAS): a simple method for the assessment of palliative care patients. J Palliat Care. 1991;7(2):6–9. 1714502

[23] de Graaf E, Zweers D, de Graeff A, Daggelders G, Teunissen SCCM. Does age influence symptom prevalence and intensity in hospice patients? A retrospective cohort study. Journal of Geriatrics and Palliative Care. 2014;S(1):7.

[24] Johnson MH. How does distraction work in the management of pain? Curr Pain Headache Rep. 2005;9(2):90–5. 15745617 10.1007/s11916-005-0044-1

[25] Konishi S, Kitagawa G. Information criteria and statistical modeling. Springer Series in Statistics, Springer, New York ; 2008.

[26] Breheny P, Burchett W. Visualization of regression models using visreg. R J. 2017;9(2):56–71.

[27] Mandrekar JN. Receiver operating characteristic curve in diagnostic test assessment. J Thorac Oncol. 2010;5(9):1315–6. 20736804 10.1097/JTO.0b013e3181ec173d

[28] Stevenson M, Sergeant E. epiR: Tools for the Analysis of Epidemiological Data. R package version 2.0.60. 2023.Available at: https://cran.r-universe.dev/epiR

[29] IBM: IBM SPSS Statistics 23. 2016;23: https://www.ibm.com/support/pages/downloading-ibm-spss-statistics-23. Accessed 7 Nov 2020.

[30] R Core Team: R: A Language and Environment for Statistical Computing. R Foundation for Statistical Computing, Vienna. 2021, Version 2.0.60. Available at:https://www.R-project.org

[31] Zaken MvA. Voldoen aan de Algemene verordening gegevensbescherming (AVG) - Privacy en persoonsgegevens - Rijksoverheid.nl. 2017. Available at: https://www.rijksoverheid.nl/onderwerpen/privacy-en-persoonsgegevens/voldoen-aan-de-avg. Accessed 7 Nov 2020.

[32] World Medical Association. World Medical Association Declaration of Helsinki: ethical principles for medical research involving human subjects. JAMA. 2013;310(20):2191–4. 10.1001/jama.2013.281053 24141714

[33] Koninkrijksrelaties MvBZe. Wet medisch-wetenschappelijk onderzoek met mensen. Available at: https://wetten.overheid.nl/BWBR0009408/2020-01-01. Accessed 7 Nov 2020.

[34] van Lancker A, Velghe A, van Hecke A, Verbrugghe M, van den Noortgate N, Grypdonck M, Verhaeghe S. Prevalence of symptoms in older cancer patients receiving palliative care: A systematic review and Meta-Analysis - ScienceDirect. J Pain Symptom Manag. 2014;47(1):90–104. 10.1016/j.jpainsymman.2013.02.016 23764109

[35] Helme RD, Gibson SJ. The epidemiology of pain in elderly people. Clin Geriatr Med. 2001;17(3):417–31. 11459713 10.1016/s0749-0690(05)70078-1

[36] Chen MC, Chang C, Glover GH, Gotlib IH. Increased insula coactivation with salience networks in insomnia. Biol Psychol. 2014;97:1–8. 24412227 10.1016/j.biopsycho.2013.12.016 PMC3961550

[37] Dobryakova E, DeLuca J, Genova HM, Wylie GR. Neural correlates of cognitive fatigue: cortico-striatal circuitry and effort-reward imbalance. J Int Neuropsychol Soc. 2013;19(8):849–53. 23842042 10.1017/S1355617713000684

[38] Labrakakis C. The role of the insular cortex in pain. Int J Mol Sci. 2023;24(6):5736. 36982807 10.3390/ijms24065736 PMC10056254

[39] Pieretti S, Di Giannuario A, Di Giovannandrea R, Marzoli F, Piccaro G, Minosi P, et al. Gender differences in pain and its relief. Ann Ist Super Sanita. 2016;52(2):184–9. 27364392 10.4415/ANN_16_02_09

[40] Tinnirello A, Mazzoleni S, Santi C. Chronic pain in the elderly: mechanisms and distinctive features. Biomolecules. 2021;11(8):1256. 34439922 10.3390/biom11081256 PMC8391112

[41] Zimney KJ, Louw A, Roosa C, Maiers N, Sumner K, Cox T. Cross-sectional analysis of generational differences in pain attitudes and beliefs of patients receiving physical therapy care in outpatient clinics. Musculoskelet Sci Pract. 2022;62:102682. 36332332 10.1016/j.msksp.2022.102682

[42] Bell T, Franz CE, Kremen WS. Persistence of pain and cognitive impairment in older adults. J Am Geriatr Soc. 2022;70(2):449–58. 34741304 10.1111/jgs.17542 PMC8821128

[43] Koffel E, McCurry SM, Smith MT, Vitiello MV. Improving pain and sleep in middle-aged and older adults: the promise of behavioral sleep interventions. Pain. 2019;160(3):529–34. 30562269 10.1097/j.pain.0000000000001423 PMC6377323

[44] Yang Y, Huang R, Grol-Prokopczyk H, Torres JM. Social network change after new-onset pain among middle-aged and older European adults. Soc Sci Med. 2022;310:115215. 36054986 10.1016/j.socscimed.2022.115215 PMC9514133

[45] Jaussent I, Dauvilliers Y, Ancelin M, Dartigues J, Tavernier B, Touchon J, Ritchie K, Besset A. Insomnia symptoms in older adults: associated factors and gender differences. Am J Geriatr Psychiatry. 2011;19(1):88–97. 20808113 10.1097/JGP.0b013e3181e049b6 PMC3179987

[46] Borneman T, Koczywas M, Sun VC, Piper BF, Uman G, Ferrell B. Reducing patient barriers to pain and fatigue management. J Pain Symptom Manage. 2010;39(3):486–501. 20303026 10.1016/j.jpainsymman.2009.08.007 PMC2844345

[47] Hugel H, Ellershaw JE, Cook L, Skinner J, Irvine C. The prevalence, key causes and management of insomnia in palliative care patients. J Pain Symptom Manage. 2004;27(4):316–21. 15050659 10.1016/j.jpainsymman.2003.09.010

